# Disassembly of HIV envelope glycoprotein trimer immunogens is driven by antibodies elicited via immunization

**DOI:** 10.1126/sciadv.abh2791

**Published:** 2021-07-28

**Authors:** Hannah L. Turner, Raiees Andrabi, Christopher A. Cottrell, Sara T. Richey, Ge Song, Sean Callaghan, Fabio Anzanello, Tyson J. Moyer, Wuhbet Abraham, Mariane Melo, Murillo Silva, Nicole Scaringi, Eva G. Rakasz, Quentin J. Sattentau, Darrell J. Irvine, Dennis R. Burton, Andrew B. Ward

**Affiliations:** 1 Department of Integrative Structural and Computational Biology, The Scripps Research Institute, La Jolla, CA 92037, USA.; 2 Consortium for HIV/AIDS Vaccine Development (CHAVD), The Scripps Research Institute, La Jolla, CA 92037, USA.; 3 Department of Immunology and Microbiology, The Scripps Research Institute, La Jolla, CA 92037, USA.; 4 International AIDS Vaccine Initiative–Neutralizing Antibody Center (IAVI-NAC), The Scripps Research Institute, La Jolla, CA 92037, USA.; 5 Koch Institute for Integrative Cancer Research, Massachusetts Institute of Technology, Cambridge, MA 02139, USA.; 6 Wisconsin National Primate Research Center, University of Wisconsin-Madison, Madison, WI 53715, USA.; 7 Sir William Dunn School of Pathology, University of Oxford, Oxford, OX1 3RE, UK.; 8 Department of Biological Engineering, Massachusetts Institute of Technology, Cambridge, MA 02139, USA.; 9 Howard Hughes Medical Institute, Chevy Chase, MD 20815, USA.; 10 Ragon Institute of MGH, MIT and Harvard, Cambridge, MA 02139, USA.

## Abstract

Rationally designed protein subunit vaccines are being developed for a variety of viruses including influenza, RSV, SARS-CoV-2, and HIV. These vaccines are based on stabilized versions of the primary targets of neutralizing antibodies on the viral surface, namely, viral fusion glycoproteins. While these immunogens display the epitopes of potent neutralizing antibodies, they also present epitopes recognized by non-neutralizing or weakly neutralizing (“off-target”) antibodies. Using our recently developed electron microscopy polyclonal epitope mapping approach, we have uncovered a phenomenon wherein off-target antibodies elicited by HIV trimer subunit vaccines cause the otherwise highly stabilized trimeric proteins to degrade into cognate protomers. Further, we show that these protomers expose an expanded suite of off-target epitopes, normally occluded inside the prefusion conformation of trimer, that subsequently elicit further off-target antibody responses. Our study provides critical insights for further improvement of HIV subunit trimer vaccines for future rounds of the iterative vaccine design process.

## Teaser

HIV Env trimers elicit antibodies that lead to their own destruction.

## INTRODUCTION

HIV infects approximately 1.7 million individuals each year, with an estimated 38 million currently living with the virus (unaids.org). While there are a large number of antiretroviral therapies available, a vaccine would be the most effective measure for controlling the spread of the virus and best hope for eventual eradication. To that end, there are a number of parallel efforts being developed at preclinical and clinical stages that use a stable, soluble HIV envelope glycoprotein (Env) trimer as a protein subunit immunogen (*1*). In preclinical animal models, variants of these trimers have elicited consistent autologous and sporadic heterologous neutralizing antibody responses against difficult-to-neutralize tier 2 viruses (*2*–*5*). Although these results are encouraging, there is still much work to be done to elicit broad and potent immune responses. To this end, there are intensive efforts to understand the types of immune responses elicited by various trimer vaccine candidates in great detail. Hence, elucidating on- and off-target (neutralizing and non-neutralizing) antibody responses elicited by these trimers remains a critical component for immunogen redesign and the iterative, rational vaccine design process (*6*, *7*).

Env is an inherently metastable protein because it must undergo drastic conformational changes to drive entry of the virus into the host cell. Known broadly neutralizing antibody (bnAb) epitopes are only present on the prefusion conformation of Env, and therefore, rational vaccine design is focused on producing this form of Env (*1*, *8*). Most of the current soluble Env trimer immunogens are based on the SOSIP design that includes a disulfide bond, which links gp120 to gp41 and an isoleucine-to-proline mutation in the heptad repeat 1 region that stabilizes the prefusion conformation of the trimer (*3*, *9*–*11*). Further, truncating the gene at residue 664 improves solubility and monodispersity of the trimer (*12*). These trimers are designed to antigenically mimic native envelope trimers (Env), including glycosylation (*13*–*16*), while also being soluble and highly stable. Soluble HIV Env trimer immunogen design has increased trimer stabilization to above 75°C (*17*). These highly stable trimers mask internal and undesirable epitopes exposed on gp120 or non-native versions of Env and exhibit an antigenic profile that preferentially exposes neutralizing epitopes and not non-neutralizing, and otherwise immunodominant, epitopes (*18*).

Despite being highly stable with antigenically optimized surfaces, the HIV Env trimers described above still elicit strong non-neutralizing antibody responses (*6*, *7*, *18*). Hence, the trimers require iterative improvement via testing in animals, characterization of elicited antibodies, mapping of epitopes, and further redesign. To accelerate this process, we recently developed electron microscopy polyclonal epitope mapping (EMPEM), which is a relatively high-throughput and comprehensive assay for mapping antibody responses in polyclonal sera (*6*). To conduct EMPEM studies, immunoglobulins from the sera or plasma of infected or vaccinated animals are isolated, made into complexes with protein subunit vaccines, and then imaged and reconstructed using single-particle electron microscopy (*6*). Because of the speed and information content generated by EMPEM, it has become an increasingly valuable tool for informing immunogen redesign, particularly for identifying on- and off-target antibody responses (*7*).

We previously showed that base-binding antibodies appeared after the priming immunization in rabbits and were present, typically at increasing abundance, after subsequent booster immunizations (*6*). Immune responses to soluble Env trimer vaccination have also revealed an abundance of elicited base-binding antibodies (*4*, *7*, *19*). Some estimates for base-directed responses are greater than 90%, which is a highly unproductive skewing of antibodies away from the relevant neutralizing epitopes (*18*). Recently, Moyer *et al.* (*20*) attached Env trimers to alum via a C-terminal phosphoserine tag at the base of the trimer in an attempt to mask off-target base antibodies in rabbits. EMPEM analysis revealed that the alum-trimer complex did not completely prevent base responses, although there was a greater diversity of antibodies bound to neutralizing epitopes outside the base, particularly at the earlier time points. These data suggest that blunting of the base response early on increased the diversity of epitopes targeted, including neutralizing epitopes (*20*). Other attempts to mask the base via attachment of Env trimers to nanoparticles have only had limited success (*21*).

While the trimer base is typically an irrelevant and undesirable epitope, a few bnAbs targeting an epitope near the base have been described. Monoclonal antibody (mAb) 35O22 binds parallel to the viral membrane at the base along a conserved region at the gp120/gp41 interface and neutralizes 62% of 181 pseudoviruses with median inhibitory concentration (IC_50_) < 50 μg/ml (*22*). Glycans located near the gp120/gp41 interface, including N88, are among the most conserved on Env (*22*). Antibodies such as 3BC315 and 1C2 bind at the gp120/gp41 interface to an overlapping but unique epitope from 35O22. Notably, binding of these bnAbs causes the Env trimer to fall apart into its protomer components (*4*, *23*) by disrupting the tryptophan clasp that stabilizes the base of the trimer (*24*). In the past, EMPEM was used only to identify epitopes on Env trimers, so protomeric classes were not included in analysis. Here, we emphasize that non–Env-like classes should also be scrutinized alongside the routine epitope identification on Env trimers. All of the known neutralizing base antibodies approach the bottom of the trimer from an angle roughly parallel to the membrane, while the non-neutralizing antibodies approach the base at a steeper angle that would clash with the membrane.

While trimer degradation is a potential mechanism for viral neutralization, it is confined to a small subset of antibodies described above that meet specific criteria. Here, we demonstrate that there are also classes of base-directed, non-neutralizing antibodies that cause soluble Env trimers to disassemble into protomers. Almost all Env trimers that we have examined degrade in this manner, and we have observed this phenomenon using sera from both rabbits and nonhuman primates. Most perniciously, secondary antibody responses to internal, non-neutralizing epitopes demonstrate that antibody-induced trimer degradation happens in vivo and may further thwart productive immune responses.

## RESULTS

### Highly stabilized Env trimers can degrade into component protomers

**Fig. 1:**
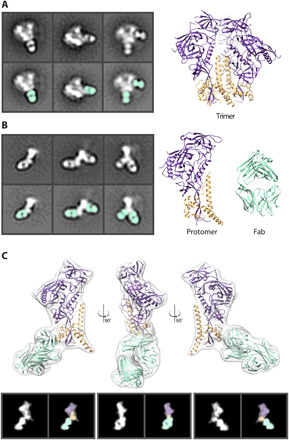
HIV Env protein in nsEM. (**A**) Negative stain 2D classes of Env trimer with Fabs bound. Fabs are highlighted in seafoam green. Cryo–electron microscopy (cryo-EM)–derived model of Env BG505 SOSIP trimer (PDB: 6DID). gp120 (purple) and gp41 (orange). (**B**) Negative stain 2D class of Env protomers with Fabs bound. Models of a single Env protomer and Fab [Protein Data Bank (PDB): 6DID] in seafoam green are shown as high-resolution models for comparison. (**C**) Low-pass–filtered cryo-EM map of an Env protomer bound to 1C2 Fab (PDB: 6P65) were used to generate 2D back projections, which clearly reveal the gp120 region as a wider density at the apex. This easily identifiable topology in the 2D classes enables assignment of the orientation of protomer and subsequent identification of epitopes to be determined.

Single-particle negative stain electron microscopy (nsEM) is a simple and quick technique to assess sample purity and map antibody epitopes on viral glycoproteins (*6*, *25*–*28*). This technique has now become commonplace for evaluating the homogeneity and native-like conformation of Env trimers being developed as protein subunit immunogens. In this study, we conducted EMPEM using various serum antibodies elicited by different stabilized Env trimers. Within each set of two-dimensional (2D) classes, we observed a range of phenotypes including intact trimers bound to antibody fragments antigen binding (Fabs) (Fig. 1A) and monomeric Env, or protomers, bound to Fabs (Fig. 1B). These protomers are compact and appear to remain in the prefusion conformation, likely because of the engineered stabilizing mutations that include a disulfide bond that links gp120 to gp41 and an isoleucine-to-proline mutation in the heptad repeat region of gp41 that prevents the conformational transition into an extended helix (*3*, *9*–*11*), as well as others. Given the relative abundance of protomeric species in different Env trimer samples imaged for EMPEM analysis, we undertook a systematic study to further investigate the causes and effects of trimer degradation into protomers.

### Some base-binding antibodies cause trimer degradation

**Fig. 2:**
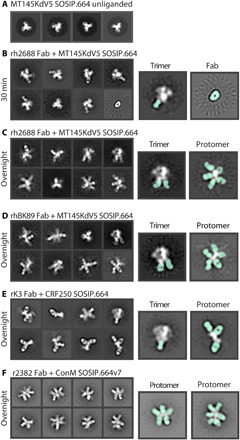
Env degradation after polyclonal Fab incubation. (**A**) Negative stain 2D classes of unliganded MT145KdV5 SOSIP.664 Env trimer. Mostly top views because of lack of tumbling. (**B**) Polyclonal Fab from rhesus macaque 2688 in complex with MT145dV5 SOSIP.664 trimer incubated for 30 min and placed on an nsEM grid. Highlighted classes show intact trimer with Fab bound (seafoam green) (left) and unliganded Fab (right). (**C**) Trimer stays intact within 30 min of incubation but will degrade over time, allowing secondary Fabs to bind internal neo-epitopes of the protomer. (**D**) Polyclonal Fab from rhesus macaque BK89 in complex with MT145dV5 SOSIP.664 shows a similar outcome as with previous rhesus macaque polyclonal serum. (**E**) Rabbit-derived polyclonal Fab in complex with CRF250 SOSIP.664 Env shows trimer degradation and base-binding Fabs bound to a few intact trimers. (**F**) Rabbit-derived polyclonal Fab in complex with ConM SOSIP.664v7 Env showing highly decorated Env protomers. Trimer class is not observed.

Before immunogenicity, antigenicity, or high-resolution structural studies, we routinely evaluate Env trimers by single-particle nsEM to ensure homogeneous distribution of prefusion conformation trimer (Fig. 2A and fig. S1). For epitope mapping, we typically incubate trimers overnight with an excess of Fab (either monoclonal or polyclonal), and the resulting complexes are purified using size exclusion chromatography and deposited onto nsEM grids.

Using Env trimers of various subtypes and polyclonal Fabs from different animal models immunized with soluble Env trimers (rabbits and macaques), we observed a range of trimeric and protomeric Env-Fab complexes (Fig. 2, B to F, and figs. S2 to S6, S9, and S17). Consistent with previous observations, we noted a large percentage of trimers with Fabs bound to the immunodominant base epitope (Fig. 2, B to F). The extended structure and globular gp120 head of the protomer was readily distinguishable in the 2D images, enabling relative locations of bound Fabs to be delineated (Fig. 1C). We also observed 2D classes of protomers that were highly decorated with Fabs (Fig. 2, C to F). Notably, in nearly all 2D class averages of protomers, there was a Fab bound to the base (Fig. 2, B to F).

In some studies, we observed almost no intact trimers with overnight incubation, although shorter incubation times with Fabs (30 to 60 min) could be used to increase the percentage of intact trimers. When rh2688 polyclonal Fabs were added to the MT145KdV5 SOSIP.664 Env trimer (*27*) for 30 min and then deposited on an EM grid, base-binding antibodies bound to trimers were visible in 2D classes. We did not detect protomers under these experimental conditions, although they may have been present at very low abundance (Fig. 2B). For comparison, the same sample, when incubated overnight, resulted in extensive trimer disassembly (Fig. 2C). Notably, trimers alone when incubated overnight do not decompose into protomers (Fig. 2A). These observations are consistent with a mechanism in which antibody binding drives the degradation process.

Close inspection of some 2D images revealed that Fabs were bound to both sides of the Env protomer (Fig. 2, C, D, and F, and fig. S12), suggesting that at least some Fabs recognized epitopes not exposed on an intact Env trimer. Many published and unpublished examples of Env bound to multiple Fabs show a maximum of three antibodies per protomer. Therefore, observing four or more Fabs on a single protomer is consistent with additional Fabs bound to internal epitopes not accessible on the trimeric form of Env. For B cells to gain access to those off-target neo-epitopes and generate an antibody response, the trimer must therefore degrade in vivo. While the 2D images are quite revealing, we could not generate stable 3D reconstructions of the protomers, consistent with a high degree of heterogeneity and limited views.

### Antibody-dependent trimer degradation increases over the course of vaccination

**Fig. 3:**
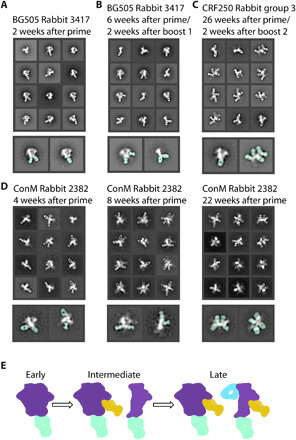
Antibody-dependent trimer degradation occurs over time. (**A**) Two weeks after initial injection of antigen, the immune system develops base-binding antibodies but Env stays intact. (**B**) Six weeks after prime and 2 weeks after a boost, the immune system develops new glycan hole peptide region antibodies and base-binding antibodies that cause trimer degradation. (**C**) Twenty-six weeks after initial injection, rabbit serum causes complete degradation of trimer with Fabs bound to all sides of the protomer. Highlighted classes show location of Fab (seafoam green). (**D**) Longitudinal study of a single rabbit during an immunization trial shows early base-binding antibodies to trimers that precedes increasing levels of trimer disassembly. By 22 weeks after prime, fully decorated protomers are the most common species observed in the 2D classes. (**E**) Early time points show immediate base response (seafoam green). As time goes on, other potentially neutralizing antibodies appear (mustard yellow). At the same time, the base response causes degradation of the Env trimer, resulting in off-target secondary responses (blue).

In our previous EMPEM study of serial vaccinations in rabbits, we showed a progression in antibody responses starting with base-binding antibodies, with some animals going on to develop neutralizing antibodies to the glycan hole epitope (*25*). On the basis of the observations described above, we went back and reanalyzed these data to investigate whether trimer degradation also occurred in that experiment. We note that our original 3D image reconstruction workflow would have excluded nontrimeric forms of Env. Because we had collected data after the priming and each of the boosts, we were also able to monitor how trimer degradation evolved over time. Two weeks after the priming vaccination using BG505 SOSIP.664 Env, nsEM showed either unliganded Env or base-binding antibodies bound to trimer (Fig. 3A and fig. S7). At 6 weeks after immunization and 2 weeks after a boost, nsEM showed base-binding antibodies and protomers with Fabs bound (Fig. 3B and figs. S8 and S17). Another example of a longitudinal study shows the progression of trimer degradation. At 4 weeks after immunization, rabbit polyclonal Fabs bind to various epitopes on the Env trimer including the base. At 8 weeks after prime, Fabs bound to protomers were observed with week 22 after prime, showing almost exclusively protomer/Fab complexes (Fig. 3D and figs. S10 to S12). A cartoon depiction (Fig. 3E) shows the progress of trimer disassembly in vivo. Base-binding antibodies appear first but may not necessarily cause the trimer to disassemble, as those antibodies are often still visible on intact Env trimers in later time points (Figs. 2, B and C, and 3C and fig. S9). As the immune response progresses, base-binding antibodies bind at higher stoichiometry and drive the trimer disassembly phenotype. The resulting protomers then present internal epitopes to the immune system and elicit additional off-target responses. In some cases, the secondary antibody responses are quite extensive, and five to six Fabs can be visualized bound to a single protomer (Figs. 2, C and D, and 3, B to D, and fig. S9). These undesirable epitopes, which do not contain any glycans, appear highly immunogenic and may therefore distract the immune system from focusing on the more desirable protective broadly neutralizing epitopes.

### Broadly neutralizing monoclonal antibodies can also cause trimer degradation

**Fig. 4:**
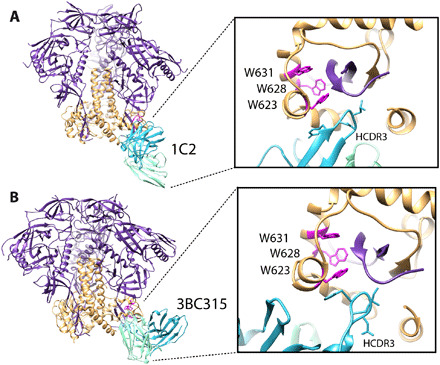
Trimer degradation caused by monoclonal antibodies. (**A**) Structure of 1C2 antibody bound to 16055 NFL TD 2CC + Env gp120 in purple and gp41 in orange (PDB: 6P65). (**B**) Cryo-EM–derived structure of BG505 SOSIP.664 in complex with 3BC315. Zooming in on the epitope/paratope reveals a long HCDR3 (blue) wedged up into the base of the trimer that disrupts the tryptophan clasp (magenta), which is the likely mechanism of degradation.

**Fig. 5:**
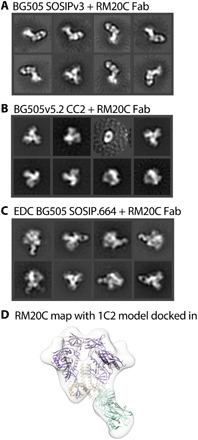
Trimer degradation-causing Fab in complex with BG505 variants. (**A**) nsEM 2D classes show that BG505 SOSIPv3 degrades into protomers when incubated overnight with RM20C. (**B**) Adding a CC mutation prevents binding of the RM20C Fab all together. (**C**) Chemically cross-linking EDC BG505 SOSIP.664 keeps trimer intact when RM20C binds. (**D**) nsEM-derived map of RM20C in complex with EDC BG505 SOSIP.664. Model of 1C2 (PDB: 6P65, purple and light orange) docked in with PDB: 6PEH (seafoam green) docked in as Fab.

In previous studies, we also observed timer degradation into protomers in the presence of the bnAbs 3BC315 and 1C2 (*4*, *29*). Both antibodies bind the base of the Env trimer and interact with gp120 and gp41 (Fig. 4). A long HCDR3 wedges itself into the gp120/gp41 interface and disrupts the tryptophan clasp region. This insertion causes a conformational change in gp41 that leads to destabilization and degradation. RM20C, which was recently isolated from a BG505 SOSIP.664–immunized macaque, binds to the base of the trimer and causes degradation into protomers when incubated overnight (Fig. 5A and fig. S13). RM20C does not neutralize BG505 virus as an immunoglobulin G (IgG) but can weakly neutralize as a Fab, consistent with the hypothesis that the disposition of the second Fab arm and Fc can sterically inhibit access to the base epitope on intact virions (*26*). Therefore, although the base epitope shared by two known bnAbs and one autologous neutralizing Fab is a bona fide site of vulnerability, access is highly constrained. Binding to this epitope in the context of the soluble trimer immunogen does not have the same constraints. Thus, non-neutralizing antibodies that approach the trimer at a steeper angle than 3BC315 and 1C2, which approach parallel to the membrane, or have the heavy chain of the antibody positioned lower than the light chain, which is likely the case for RM20C, are most likely the driving force in polyclonal serum that causes trimer degradation (*26*).

### Additional disulfide bonds and chemical cross-linking prevent trimer degradation

While antibody-induced trimer degradation appears to be a problem for many of the current Env trimers being used for immunogenicity studies, there are versions of the trimer resistant to this phenotype. We recently demonstrated that an advanced version of the ConM SOSIP trimer (ConM SOSIP.664v9) that includes an engineered interprotomer disulfide bond between residues 72 and 564 resists serum-induced degradation that was observed in the parental ConM SOSIP.664v7 (*21*). An interprotomer disulfide between residues 501 and 663 of the 16055 Env was also recently reported to prevent native flexibility linked (NFL) trimer degradation induced by bnAbs 3BC315 or 1C2 (*4*, *30*). This disulfide bond is an alternative to the traditional 501 to 605 disulfide present in previously described SOSIP trimers. Here, we introduced this disulfide bond into the fully cleaved version of the BG505 Env trimer (referred to as BG505.664v5.2 CC2). The CC2 disulfide trimer knocked out binding to RM20C Fab (Fig. 5B and fig. S14).

Chemical cross-linking is another potential remedy for trimer disassembly. To test this, we first incubated BG505 SOSIP.664 (Fig. 5A and fig S13) with mAb RM20C, known to cause trimer degradation. This BG505 SOSIP.664 trimer readily degrades into protomers, while trimers that had been cross-linked using 1-ethyl-3-(3-dimethylaminopropyl)carbodiimide (EDC) (*31*) remained resistant to disassembly (Fig. 5, C and D, and figs. S15 and S16) when bound by RM20C.

## DISCUSSION

On the surface of the virion, Env is membrane bound. Despite close juxtaposition to the membrane, epitopes near the base of the trimer (e.g., 3BC315, 35022, and 1C2) (*4*, *22*, *23*) and the highly conserved membrane proximal external region (*32*, *33*) are accessible to neutralizing antibodies. Soluble Env trimers truncated at residue 664 expose a large peptidic surface at the base of the trimer that is not accessible on membrane-bound Env. Without the membrane or gp41 glycans in the way, the exposed base has emerged as an easy target for antibodies and therefore of the immunodominant response in different animal models (*6*, *7*, *18*, *19*).

Given the high thermal stability of these trimers and elicitation of neutralizing antibodies, it has been assumed that the trimers stay intact in vivo. Here, we show that, in addition to being off-target and immunodominant, base-directed responses that bind at or around the Trp clasp induce disassembly of the trimer into protomers. Somewhat ironically, this immunodominant response targets what can be a bnAb epitope but one that is highly sensitive to heavy/light-disposition. Dissociated protomers then become immunogenic in their own right, eliciting a second wave of non-neutralizing antibodies that target epitopes that reside inside the native trimer. As these epitopes are relatively large continuous peptidic surfaces devoid of glycans, like the base of the trimer, they are easier targets for recognition by B cells than the highly glycosylated exterior surface of the trimer. Thus, the immunogenicity of the trimer base represents a doubly confounding effect on neutralizing antibody responses to the trimer. While trimer degradation has occurred in all trimers that we have analyzed so far, the susceptibility and timing of degradation varied by subtype and design. Some, such as BG505 SOSIP.664, are less susceptible, while others, such as ConM SOSIP.664 v7, MT145KdV5, or CRF250 SOSIP.664 (*27*, *34*), are much more prone to antibody-dependent degradation. Antibody-induced degradation of trimers may also have implications for binding and antigenicity studies such as Octet or enzyme-linked immunosorbent assay and may therefore confound interpretation.

Encouragingly, Env trimer constructs that contain interprotomer disulfide bonds that are resistant to antibody-dependent degradation already exist (*21*). Additional disulfides may provide further stability, although these will need to be tested in immunogenicity studies. Thus, secondary immune responses to internal protomer epitopes may not be an issue, provided that more advanced designs are used. Currently, all of the Env trimers that are most advanced in preclinical or clinical programs (ClinicalTrials.gov: NCT03699241, NCT04177355, and NCT03783130) do not have these stabilizing disulfides, and we predict that humans will therefore also produce base-directed trimer degrading antibodies. Chemically cross-linked trimers, which we demonstrated are resistant to disassembly, are also being tested in humans by the European AIDS Vaccine Initiative (eavi2020.org).

Attempts to mask the base of the trimer via attachment to nanoparticles or alum have only had limited impact on reducing base-directed antibodies (*20*, *21*). Thus, further development of these strategies will be required, likely to include interprotomer disulfide bonds. There is also the potential for prime boost immunization strategies using Env trimers of different sequences at the base such that they cannot be recognized (and degraded) by the base-directed antibodies elicited by the prior immunization. Because the base of the trimer is typically shielded by the membrane, it has a relatively high degree of sequence conservation, which makes this strategy less viable. Hence, further mutating exposed base residues, or resurfacing, may be required for such an approach to work. Display of Env trimers on liposomes or delivered via nucleic acid and expressed on cell surfaces in vivo may also limit the base-directed responses.

In summary, our study reveals a previously underappreciated phenomena of base-directed antibody-induced in vivo Env subunit trimer degradation and secondary antibody responses. These phenomena are unlikely to be unique to HIV Env. Given the large number of efforts to use subunit vaccines against other pathogens including influenza, respiratory syncytial virus (*35*), SARS-CoV-2, and others, it will also be important to assess antibody-mediated disassembly in those systems. EMPEM was integral to our ability to uncover this confounding factor, further demonstrating the power of single-particle EM approaches to comprehensively characterize heterogenous ensembles of proteins and protein-protein complexes. Standard single-particle EM workflows usually eliminate, or overlook, data that do not contribute to stable 3D reconstructions. Hence, we advise that future studies include comprehensive 2D and 3D classification to reveal the full extent of species in a given ensemble of Env-antibody complexes.

## MATERIALS AND METHODS

### Ethics statement

The rabbit and rhesus macaque animal studies were approved and carried out in accordance with protocols provided to the Institutional Animal Care and Use Committee, respectively, at The Scripps Research Institute (La Jolla, CA) under approval number 19-0020 and at Wisconsin Primate Center under approval number G005109. The animals at both facilities were kept, immunized, and bled in compliance with the Animal Welfare Act and other federal statutes and regulations relating to animals and in adherence to the *Guide for the Care and Use of Laboratory Animals* (National Research Council, 1996).

### Immunization and sampling

Twenty-four 12-week-old female New Zealand white rabbits were primed (week 0: CRF250 SOSIP trimer) and boosted [boost 1: week 8, CRF250 SOSIP (group-K) or BG505-CRF250V1V2 chimeric SOSIP (group-L) or its N130 (group-M) or V2′ (group-N) glycan-filled SOSIP trimers; boost 2: week 24, ZM179-ZM233V1V2 chimeric SOSIP trimer] with 50 μg of trimer protein and 375 μg of ISCOM (Immune Stimulating Complex matrix)-like saponin adjuvant in 600 μl of phosphate-buffered saline (PBS; 300-μl intramuscular injection into each hindleg). Blood was drawn from the marginal ear vein into EDTA or untreated blood collection tubes from select time points (weeks 2, 8, 10, and 26), and the polyclonal IgGs for EMPEM were purified from immune sera/plasma. All procedures were performed in anesthetized animals. Two groups of six rhesus macaques each, all females between the ages 5 and 7 years, were immunized with chimpanzee simian immunodeficiency virus MT145KdV5 SOSIP (group 1) or MT145KdV5 SOSIP conjugated with alum through pSer peptide linkers, as described previously (*20*). Animals were primed (week 0) and boosted (week 8) intramuscularly by injecting 100 μg of MT145KdV5 or MT145KdV5-pSer trimer proteins along with 1 mg of alum and 375 μg of Saponin and MPLA (monophosphoryl lipid A) nanoparticle (SMNP) adjuvant per animal per immunization. Vaccine regimens for these studies are summarized in the Supplementary Materials (fig. S17). EDTA blood at select time points was collected from animals for isolating polyclonal IgGs for EMPEM analysis.

### Monoclonal Fab production

RM20C Fab was expressed in human embryonic kidney (HEK) 293F cells and purified using affinity chromatography. Briefly, HEK293F cells (Invitrogen) were cotransfected with heavy and light-chain plasmids (1:1 ratio) using PEImax. The transfection was performed according to the manufacturer’s protocol. The supernatant was harvested 6 days following transfection and passed through a 0.45-μm filter before purification using CaptureSelect CH1-XL (Thermo Fisher Scientific) affinity chromatography.

### Env protein production

To produce the BG505-CC2 construct, the following mutations were introduced into the BG505 SOSIPv5.2 construct (*17*) using the QuikChange Lightning Multi Site-Directed Mutagenesis Kit (Agilent): P240T, S241N, M271I, F288L, T290E, P291S, R500A, L568D, V570D, R585H, C605T, S613T, Q658T, and L663C. Env trimers were expressed in 293F cells and purified using PGT145 affinity chromatography as described previously (*17*).

### Env controls and monoclonal complexes

Unliganded Env trimers were deposited directly onto nsEM grids as controls. Env trimers were incubated overnight at room temperature with monoclonal Fabs in excess of 4.5 M. Complexes were diluted to 0.03 mg/ml and deposited onto nsEM grids. EDC BG505 SOSIP.664 trimer was purified and chemically cross-linked as described previously (*29*, *31*).

### Electron microscopy polyclonal epitope mapping

The polyclonal IgGs from serum or plasma were purified by incubating with equal volume of Protein A and G Sepharose resin (GE Healthcare) at 4°C overnight, followed by washing with PBS and elution of IgGs with 2 M citric acid into 0.2 M TrisBase. Eluents were buffer-exchanged into PBS using 30-kDa Amicon tube protein concentrators (MilliporeSigma). The purified polyclonal IgGs were then digested into Fabs using the Pierce Fab Preparation Kit (Thermo Fisher Scientific). Briefly, after preparation of the IgG sample using Zeba Spin Desalting Columns, 0.25 ml of equilibrated immobilized papain was incubated with 1 to 2 mg of IgG in digestion buffer rotating for 4 to 5 hours at 37°C. Digest was separated from immobilized papain by centrifugation, followed by mixing incubation with Protein A resin at room temperature for 30 min. The flow-through after centrifugation containing Fabs was then buffer-exchanged into tris-buffered saline using 10-kDa filter Amicon tubes (MilliporeSigma). For EMPEM, 500 μg of serum Fab was added to 15 μg of Env trimer and incubated overnight at 4°C. The EMPEM complex was purified from excess Fab using size exclusion chromatography. The complex peak was concentrated to about 0.03 mg/ml and deposited directly onto an nsEM grid.

### Negative stain electron microscopy

Env complexes were added to carbon-covered 400-mesh copper grids. Grids were stained with 2% UF (Uranyl Formate). Micrographs were collected using Leginon (*36*) on either a TF20 or Spirit electron microscope. They were equipped with either a TVIPS or EAGLE 4kx4k camera. Particles were picked using DoGpicker and stacked using Appion (*37*). Particles were 2D-classified by Relion (2 and 3) (*38*, *39*). Particles resembling an Env trimer or Env protomer were selected for further processing. Coloring of 2D class images was done in Photoshop. 3D maps were visualized in Chimera (*40*).

## SUPPLEMENTARY MATERIALS

Supplementary material for this article is available at http://advances.sciencemag.org/cgi/content/full/7/31/eabh2791/DC1

## Appendix

View/request a protocol for this paper from *Bio-protocol*.
